# Predicting Human Cooperation

**DOI:** 10.1371/journal.pone.0155656

**Published:** 2016-05-12

**Authors:** John J. Nay, Yevgeniy Vorobeychik

**Affiliations:** 1 School of Engineering, Vanderbilt University, Nashville, Tennessee, United States of America; 2 Department of Electrical Engineering & Computer Science, Vanderbilt University, Nashville, Tennessee, United States of America; Tianjin University of Technology, CHINA

## Abstract

The Prisoner’s Dilemma has been a subject of extensive research due to its importance in understanding the ever-present tension between individual self-interest and social benefit. A strictly dominant strategy in a Prisoner’s Dilemma (defection), when played by both players, is mutually harmful. Repetition of the Prisoner’s Dilemma can give rise to cooperation as an equilibrium, but defection is as well, and this ambiguity is difficult to resolve. The numerous behavioral experiments investigating the Prisoner’s Dilemma highlight that players often cooperate, but the level of cooperation varies significantly with the specifics of the experimental predicament. We present the first computational model of human behavior in repeated Prisoner’s Dilemma games that unifies the diversity of experimental observations in a systematic and quantitatively reliable manner. Our model relies on data we integrated from many experiments, comprising 168,386 individual decisions. The model is composed of two pieces: the first predicts the first-period action using solely the structural game parameters, while the second predicts dynamic actions using both game parameters and history of play. Our model is successful not merely at fitting the data, but in predicting behavior at multiple scales in experimental designs not used for calibration, using only information about the game structure. We demonstrate the power of our approach through a simulation analysis revealing how to best promote human cooperation.

## Introduction

The Prisoner’s Dilemma game has been a subject of extensive research due to its importance in understanding the ever-present tension between individual self-interest and social benefit [1–3]. From a theoretical perspective, a strictly dominant strategy (defection), when played by both players, is mutually harmful: cooperation by both yields significant mutual benefits relative to defection. For example, local maintenance of shared drinking water systems in rural communities represents a Prisoner’s Dilemma that can result in a “tragedy of the commons” [4]. From each community member’s perspective, they are better off if someone else invests in maintaining the infrastructure. If the majority of the community adopts this strategy, everyone is worse off because the system breaks down and no longer provides clean water.

In most social dilemma settings, however, interactions are repeated. Thus, for example, community members must repeatedly make water infrastructure investment decisions. Repetition of the Prisoner’s Dilemma, a more realistic model of human interaction than a one-shot game, can theoretically give rise to cooperation as an equilibrium if players are sufficiently “patient”; still, defection remains an equilibrium as well, and this ambiguity is difficult to resolve. In particular, theoretical treatment of repeated Prisoner’s Dilemma games is not instructive in identifying when cooperation or defection emerges as the predominant outcome. Given the limitations of theory in explaining repeated cooperation, researchers have turned to experiments to better understand behavior and the effects of institutional structure on social outcome by considering different game structures and investigating associated cooperation proclivities of human subjects [5]. The experiments highlight that humans often cooperate, but the overall level and temporal evolution of cooperation vary significantly with the specific design.

We develop a predictive model of dynamic cooperation that reliably forecasts behavior across heterogeneous game designs, and then analyze this model to tease apart the magnitude and direction of the effects of game design variables on cooperation. For this purpose we compiled data from previously analyzed repeated Prisoner’s Dilemma experiments [6–13]. We created standardized measures of the game and individual behavior across these games, and used machine learning techniques to calibrate and evaluate computational models. Our model is extremely successful in predicting individual decisions, average cooperation levels, and cooperation dynamics *in games not used for model calibration*. Moreover, we demonstrate that this synthetic model can predict the high-level quantitative and qualitative findings of the human subject experiments.

The long-term goal of this research program is to map the experimental variables onto real-world policy design factors and use model analyses to inform policies that facilitate cooperation where the underlying social structure would otherwise lead to a breakdown. For instance, how can we best design development programs that lead to sufficient voluntary maintenance of shared water systems? Is it more important to increase the potential benefits of mutual cooperation over mutual defection, or to increase the benefits of mutual cooperation over losing out by being the sole cooperator?

## Data

The data are from human subjects experiments that used real financial incentives and transparently conveyed the rules of the game to the subjects, which is standard procedure in experimental economics. Subjects anonymously interact and their decisions to cooperate or defect at each time period of each interaction are recorded. They receive payoffs proportional to the outcomes in a specified payoff table similar to Table 1. From the description of the experiments in the published papers and the publicly available data sets, we were able to build a comprehensive collection of game structures and individual decisions.

**Table 1:** Payoff table. Payoff table where one player plays from the perspective of the columns and the other from the rows. For this to be a repeated Prisoner’s Dilemma, it must hold that *T* > *R* > *P* > *S*, and *R* > (*S* + *T*)/2 [14].

	C	D
**C**	*(R,R)*	*(S,T)*
**D**	*(T,S)*	*(P,P)*

The thirty game structures that we compiled varied substantially across a number of dimensions, aside from player payoffs. In some structures, payoffs were deterministic, whereas others featured stochastic payoffs (in this case, the expected payoffs constituted the payoff structure). In some structures, players imperfectly observed their counterparts’ past actions. Another key distinction was whether or not a game had a fixed time horizon, or would terminate independently after each iteration with a fixed probability. Finally, while most games were played over a discrete sequence of iterations, some were in continuous time. We use nine variables to quantify game structure along these salient dimensions. *Risk* is an indicator of whether there is stochasticity in the payoffs [8, 10]. *Error* is the probability that the choice a player makes will be exogenously flipped [13]. *Infinite* is an indicator of whether interactions are indefinitely repeated or have a fixed length [7]. *δ* is the probability that the next period of the current paired interaction will occur in a infinitely game [11]. We used a formula, $E\left[InteractionLength\right]=\frac{1}{1-\delta }$, to compute *δ* for finitely repeated interactions; for instance, the finitely repeated interactions in [10] were all ten periods long so *δ* = 0.9. *Continuous* is an indicator of whether interactions are played in “continuous time,” rather than the standard discrete rounds [12]. *R* is the reward received if both players cooperate; *P* is the punishment received if both defect; *T* is the temptation to defect on the other; and *S* is the payoff for being a sucker by cooperating as the other defects (Table 1 illustrates the way in which the four payoff values map onto the Prisoner’s Dilemma bi-matrix representation).

To create standardized payoff measures from the *R, S, T, P* values, we used two differences between payoffs associated with important game outcomes, both normalized by the difference between the temptation to defect and being a sucker when cooperating as the other defects [15]. *r*_1_ is the normalized difference between the reward received if both players cooperate and the punishment received if both defect, $\frac{R-P}{T-S}$. *r*_2_ is the normalized difference between the reward received if both players cooperate and the payoff for being a sucker when cooperating as the other defects, $\frac{R-S}{T-S}$. Because $\frac{\partial r1}{\partial R}>0$, $\frac{\partial r1}{\partial P}<0$, $\frac{\partial r1}{\partial T}<0$, and $\frac{\partial r1}{\partial S}>0$, *r*_1_ has been used as an index of the cooperativeness of a Prisoner’s Dilemma [15, 16], while *r*_2_ is descriptive of how much better off a player will be if their opponent cooperates, rather than defects, while they themselves cooperate. Table 2 summarizes the game structures from the data sets we standardized and combined.

**Table 2:** Data summary. Summary of thirty game structures that compose the full combined data set [6–13]. BR 2006 [8] and DB 2005 [7] both also conducted one-shot games; we only describe and use their repeated game data. KSBS 2009 [10] also conducted games with partial information; we only describe and use their full information data. AM 1993 [6] also conducted games that matched humans with computers; we only describe and use the games they conducted where humans played other humans. FO 2012 [12] included one-shot games and games with very different protocols for how and when to make a choice in order to study continuous choices; we only use the “Grid treatment with n = 8 subperiods,” which they say is, “comparable to the 10-stage repeated games featured in previous laboratory studies.” DO 2009 [9] also conducted random matching of opponents; we only use their fixed matching treatments.

Error	Delta	Infinity	Continuous	Risk	r1	r2	Cooperation	Dataset
0.0000	0.900	0	0	0	0.18	0.590	0.60	BR
0.0000	0.900	0	0	1	0.18	0.590	0.35	BR
0.0000	0.900	1	0	0	0.33	0.670	0.56	DO
0.0000	0.900	0	0	1	0.33	0.830	0.31	KS
0.0000	0.900	0	0	0	0.33	0.830	0.57	KS
0.0000	0.500	1	0	0	0.18	0.530	0.10	DF
0.0000	0.750	1	0	0	0.18	0.530	0.20	DF
0.0000	0.500	1	0	0	0.39	0.740	0.18	DF
0.0000	0.750	1	0	0	0.39	0.740	0.59	DF
0.0000	0.750	1	0	0	0.61	0.950	0.76	DF
0.0000	0.500	1	0	0	0.61	0.950	0.35	DF
0.1250	0.875	1	0	0	0.20	0.600	0.34	FR
0.1250	0.875	1	0	0	0.33	0.660	0.49	FR
0.1250	0.875	1	0	0	0.43	0.710	0.59	FR
0.0000	0.875	1	0	0	0.60	0.800	0.74	FR
0.0625	0.875	1	0	0	0.60	0.800	0.78	FR
0.1250	0.875	1	0	0	0.60	0.800	0.57	FR
0.0000	0.900	0	0	0	0.25	0.583	0.43	AM
0.0000	0.875	0	1	0	0.11	0.560	0.27	FO
0.0000	0.875	0	1	0	0.14	0.710	0.33	FO
0.0000	0.875	0	1	0	0.33	0.560	0.54	FO
0.0000	0.875	0	1	0	0.43	0.710	0.62	FO
0.0000	0.500	0	0	0	0.33	0.610	0.12	DB
0.0000	0.750	0	0	0	0.33	0.610	0.24	DB
0.0000	0.750	0	0	0	0.33	0.720	0.25	DB
0.0000	0.500	0	0	0	0.33	0.720	0.13	DB
0.0000	0.500	1	0	0	0.33	0.610	0.23	DB
0.0000	0.750	1	0	0	0.33	0.610	0.35	DB
0.0000	0.750	1	0	0	0.33	0.720	0.36	DB
0.0000	0.500	1	0	0	0.33	0.720	0.31	DB

**Fig 1:**
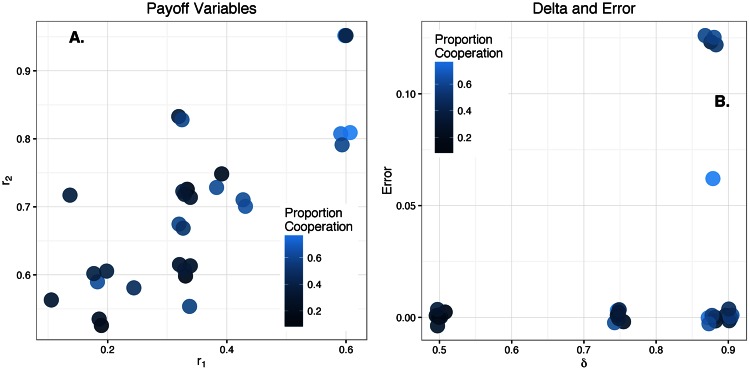
Game structures. Game structures (*n = 30*) with location based on the payoff variable values (**A**.), and delta and error values (**B**.). Colors represent proportion of cooperation observed in the game structure. Locations have been slightly randomly shifted to improve visualization.

Fig 1 plots game structures based on their values of the four quantitative game structure variables, *r*_1_, *r*_2_, *δ*, and *error*, illustrating the broad empirical support in our combined data across the values of these variables, and that there are no *simple* relationships between these variables and the proportion of cooperation that can be detected without, at least, controlling for variables not included in each plot.

**Fig 2:**
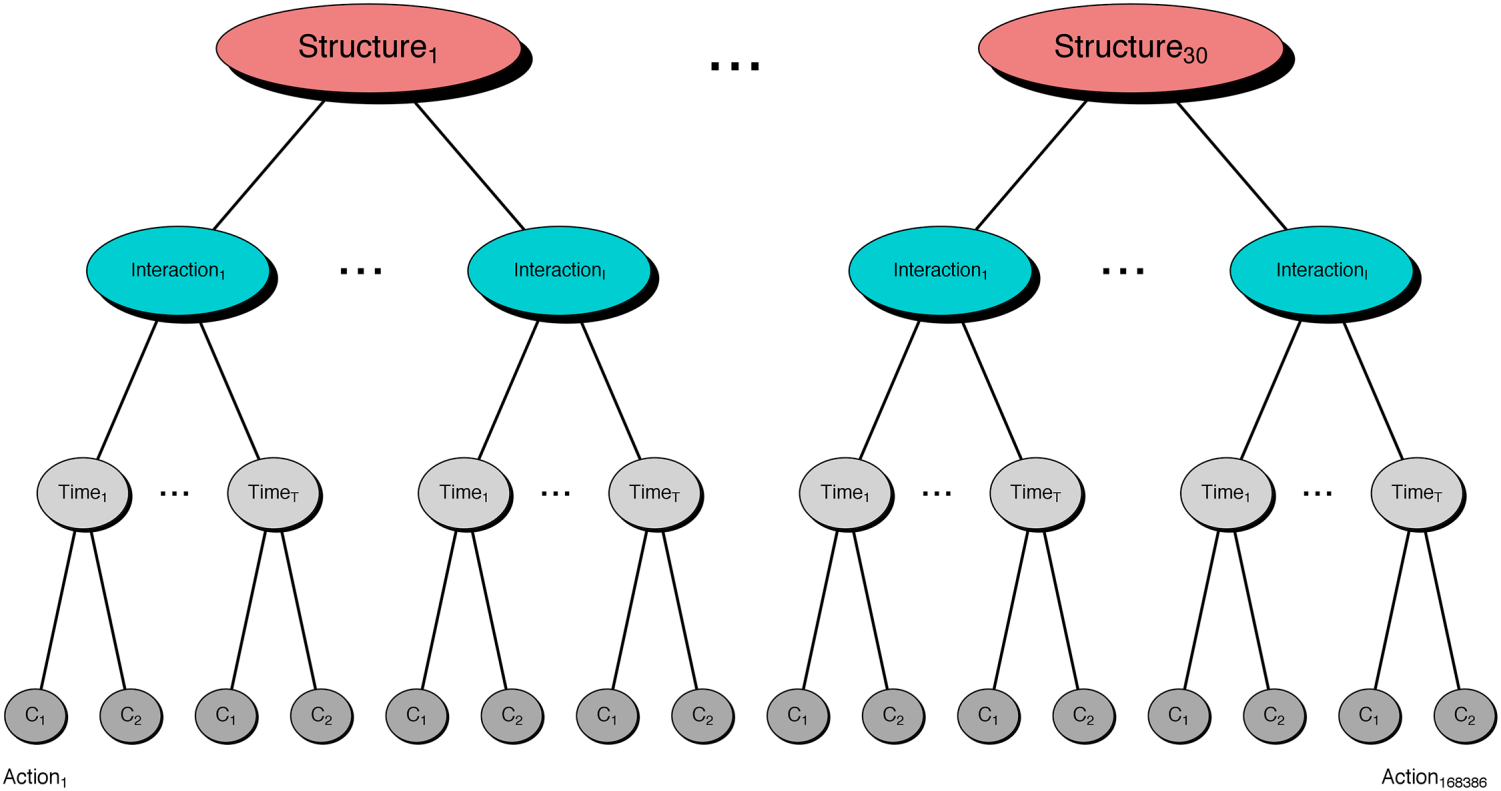
A hierarchical view of our data. The top level divides the data into 30 game structures. The next level down are the interactions between two players. Within each interaction, there are *T* time periods. In repeated games in which termination is stochastic, *T* ranges up to thirty-eight. Across all interactions and structures, *T* is five, on average. Within each time period, player 1 takes action *C*_1_ and player 2 takes action *C*_2_. 168,386 actions were taken across all the experimental data.

Our combined data set can be organized hierarchically (Fig 2). Within each game structure, there are interactions between pairs of players; these are repetitions of the same “stage-game” between the same two players. Repeating the game with past behavior as common knowledge can theoretically increase cooperation by bringing players’ reputational concerns into play. Within each interaction, there are time periods. Finally, in each time period, both players simultaneously take a single action.

Our goal was to predict behavioral patterns simultaneously at several levels within this hierarchy. Specifically, we wish to predict the effects of the game structure on average cooperation (the highest level in the hierarchy), the temporal dynamics of cooperation as a function of structure (second lowest level), and individual-level actions (lowest level). The impact of structure on cooperation has been the primary subject of experimental investigations, with the natural goal of understanding how to design institutions that promote cooperation. Understanding both short-term and long-term impacts of institutions, however, necessitates looking at behavior dynamics, rather than simply aggregate levels of cooperation. Indeed, cooperation may well be high early, but degrade with time, particularly close to the final period of the game, if it is known [8]. Finally, understanding individual behavior enables us to understand aggregate cooperation dynamics in terms of micro decision processes. If our model can successfully predict behavior at all levels in the hierarchy, we can have confidence in the ability of the resulting model to generalize experimental findings to new institutional structures, allowing us to achieve the ultimate goal: a validated computational framework for understanding and designing institutions that promote cooperation.

## Model

Our behavioral model has two parts: a “static” component that predicts a player’s first period action, and a “dynamic” component that predicts a player’s actions in subsequent times of the same interaction. Both components are logistic regressions mapping a vector of predictor variables into the probability of cooperation, with parameters learned through maximum likelihood estimation on training data. The predictor variables for first period play include the game structure, $\vec{Game}$, (*r*_1_, *r*_2_, *risk*, *error*, *δ*, *infinite*, *continuous*), and the predictors for all other time periods (i.e., the dynamic model) include the game structure, the actions of both players from the previous period, $\vec{History_{t-1}}$, and the current time period, *t*. The inclusion of the history of the interaction is motivated by evidence that most participants in repeated cooperation games condition their actions on previous play [17]. In mathematical terms, the probability of cooperation, *p*(*C*_*t*_), can be expressed as follows:

$$\begin{array}{c} p\left(C_{t}\right)=\{\begin{array}{cc} t=1 & f_{static}\left(\vec{Game}\right)\\ t>1 & f_{dynamic}\left(\vec{Game},\vec{History_{t-1}},t\right) \end{array} \end{array}$$

where *f* is determined by the logistic regression model (distinct in both cases), calibrated on behavioral data.

Specifically, we used the following equation for first period cooperation, *C*_*t* = 1_:

$$\begin{array}{c} r_{1}+r_{2}+risk+error+\delta +r_{1}\times \delta +r_{2}\times \delta +infinity+continuous \end{array}$$

We used the following equation for cooperation in periods greater than one, *C*_*t* > 1_:

$$\begin{array}{ccc} & & r_{1}+r_{2}+risk+error+\delta +r_{1}\times \delta +r_{2}\times \delta +infinity+continuous+\delta \times infinity+\\ & & \;\;my.decision_{t-1}+other.decision_{t-1}+error\times other.decision_{t-1}+t \end{array}$$

Both equations use all the structural game features, *r*_1_ + *r*_2_ + *risk* + *error* + *δ* + *r*_1_ × *δ* + *r*_2_ × *δ* + *infinity* + *continuous*, and because we hypothesized that *δ* and the payoff variables may have difference effects depending on the values of the other, we interacted them. For the dynamic model, we added an interaction term between *δ* and infinity to capture the different effect that *δ* may have when it actually determines the length of the game probabilistically. In finite games, *δ* represents a rational expectation of the length of the game from a first period perspective. In infinite games, *δ* represents a rational expectation of the length of the game for all periods. Therefore, *δ* is used as a feature in the model of first period play, and for all periods of play beyond period one there is an interaction term that multiplies the indicator variable for whether a game is infinite by the value of *δ*. We interacted error with *other*.*decision*_*t* − 1_ because the greater the value of error, the less sure the player is of the actual decision of the other player in the previous time period. The model is then a logistic sigmoid function, $\sigma \left(w^{T}X\right)=\frac{1}{1+exp(-w^{T}X)}$, acting on a linear function of these features, **X**, with a vector of weights, $\vec{w}$, the length of the feature set. The computational implementation we used was the base R “stats::glm” function and the caret “train” function [18, 19].

We compare our model’s performance to three alternative logistic regression models: “static-only,” which just uses the first component of the “full” model; “dynamic-only,” which just uses the second component; and “baseline,” which uses the observed average level of cooperation. Comparing the full model to its components allows us to understand the relative contributions of the components to its predictive power. We also compare our model to a state-of-the-art behavioral game theory model designed for forecasting play in out-of-sample games: functional experience-weighted attraction learning (fEWA) [20]. The actions available to agent *i*, which are indexed by *j*, are assumed to have numerical attractions for each time *t*, $A_{i}^{j}\left(t\right)$, and fEWA updates the attractions based on functions of *i*’s experience up to time *t* and the payoffs of the game (*i*’s chosen strategy is *s*_*i*_(*t*), *i*’s opponent’s chosen strategy is *s*_ − *i*_(*t*), *i*’s payoffs are $\pi_{i}\left(s_{i}^{j}\left(t\right),s_{-i}\left(t\right)\right)$, and *I* yields *I*(*x*, *y*) = 0 if *x* ≠ *y*, and *I*(*x*, *y*) = 1 if *x* = *y*).

$$\begin{array}{c} A_{i}^{j}\left(t\right)=\frac{\phi_{i}\left(t\right)N\left(t-1\right)A_{i}^{j}\left(t-1\right)+\left[\delta_{ij}\left(t\right)+\left(1-\delta_{ij}\left(t\right)\right)I\left(s_{i}^{j},s_{i}\left(t\right)\right)\right]\pi_{i}\left(s_{i}^{j},s_{-i}\left(t\right)\right)}{N\left(t-1\right)\phi_{i}\left(t\right)+1} \end{array}$$

Then, attractions are mapped into probabilities of choosing Cooperate or Defect the next time period with a logistic stochastic response function (see S1 Appendix for model details).

$$\begin{array}{c} P_{i}^{j}\left(t+1\right)=\frac{e^{\lambda A_{i}^{j}\left(t\right)}}{\sum_{k=1}^{m_{i}}e^{\lambda A_{i}^{k}\left(t\right)}} \end{array}$$

In order to use the empirical models of individual behavior to predict interactive outcomes of new experimental designs, we simulate discrete-time dynamic systems comprised of autonomous decision algorithms (agents) that interact with each other. This allows us to simulate the play of an experiment without any behavioral data from that experiment. Player behavior is endogenous to the simulation model, which only needs to be initialized with a game structure specification. There have been a number of studies using simulations to investigate cooperation games [21–27], and simulations have been used to inform institutional design of strategic interactions more broadly [28–30]. There has been research on cooperative equilibria models for predicting aggregate cooperation patterns [31, 32], and a significant amount of work on individual-level behavioral models [33–40]. Our work diverges from most such research in three respects: (i) agent behavior is derived solely from individual-level empirical data, and (ii) we rigorously validate our model’s ability to predict behavior by measuring performance on many unseen game structures. [27] also derive agent behavior solely from individual-level game data. However, we utilize data from many more experimental designs and from a different game.

## Results

### Individual-level performance

**Fig 3:**
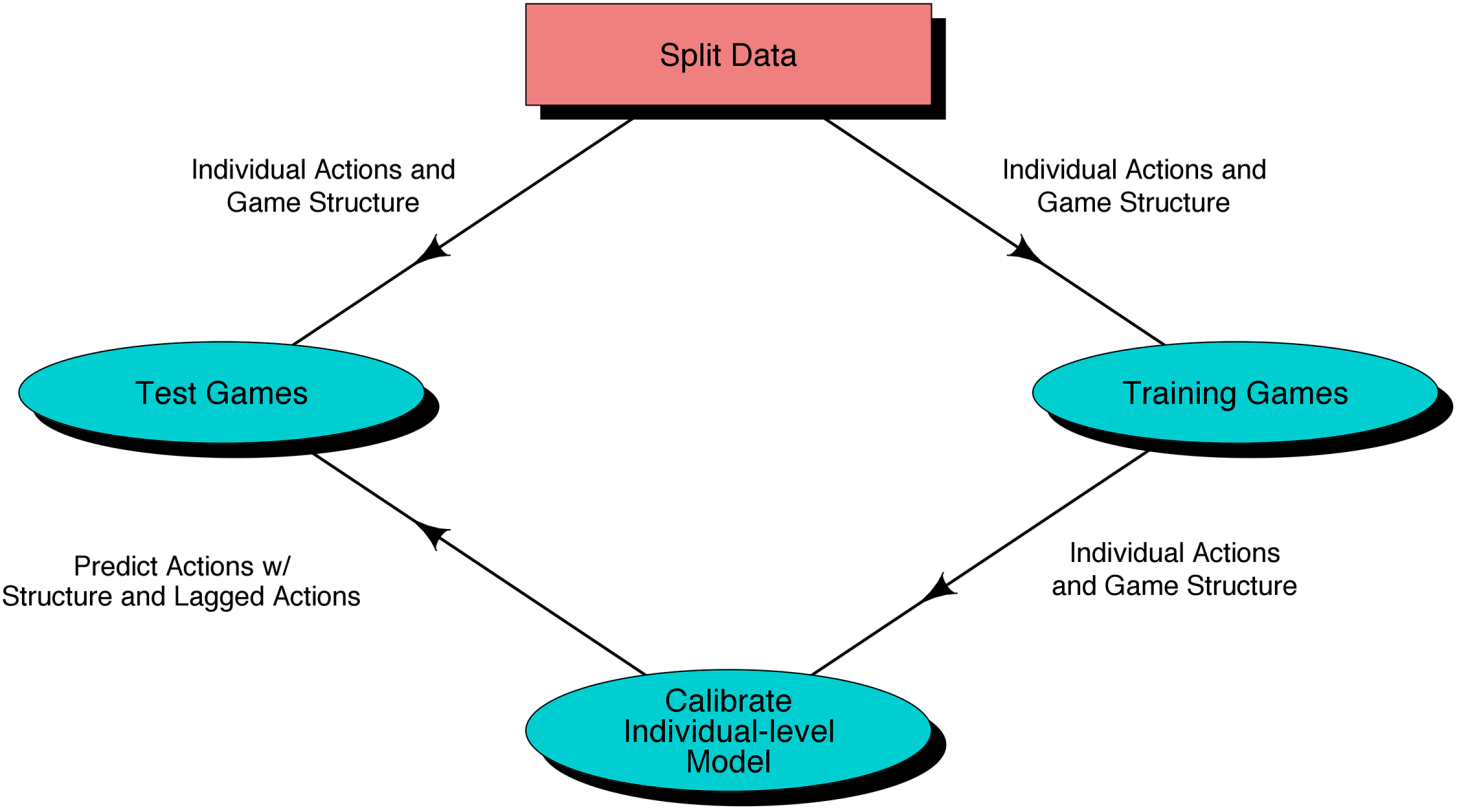
Model validation process for individual-level actions. We assign each of the thirty game structures into either training or test data. With the training data, we learn the parameters of the individual-level model, and then predict the decisions in game structures assigned to the test data. We repeat this process thirty times, including a different game structure as the held-out test each time (leave-out-one-cross-validation), until we have predictions for all the decisions for each of the game structures.

Our first investigation evaluates models’ ability to predict individual-level actions. We divide game structures into training and test groups, estimate the parameters in training game structures, and then predict actions in held-out game structures, conditioning on the game structure and the empirically observed actions of the previous period (for periods greater than one). We repeatedly execute the process, each time slightly changing the split of the data so each game structure will be in the test data once (Fig 3). The end results are out-of-sample predictions of all actions in each game structure. To make predictions with the dynamic-only model, which will have missing values for the lagged action outcomes at period one, we draw cooperate/defect actions with equal probability (corresponding, approximately, to average cooperation/defection split over all game structures). When we instead impute “period zero” outcomes as mutual cooperation the results are qualitatively the same. For this test, we measure the log-likelihood of the observed actions in the test data, given model predictions, which is a statistically proper method for evaluating the quality of probabilistic predictions. We also discretize model outputs into Cooperate or Defect to measure accuracy, which is the proportion of actions where the predicted probability of cooperation was above (below) 0.5 when the observed action was cooperation (defection).

The dynamic model performs almost as well as the full model in periods greater than one, but poorly in the first period, indeed, worse than the static model (Table 3). Overall, our relatively simple two-piece model predicts the next action a player will take with 86% accuracy on average (a remarkably good prediction, given that human behavior is generally quite noisy). Our model also significantly outperforms all alternatives in terms of the log-likelihood measure, which is more statistically appropriate in quantifying performance of stochastic forecasts, but is less intuitive.

**Table 3:** Model performance comparison. Best performance for each test is *italicized*. **First four rows** are performance on 32,614 predictions of period one actions and 135,772 predictions of period greater than one actions. Each evaluation is an average for how that model performed with out-of-sample predictions for each game structure. We conduct paired sample t-tests (not assuming equal variances) to determine if the thirty accuracy and likelihood values for the full model are statistically greater than the values of the next best model. Accuracies for *t>1* of the full model (*p = 0.03*) and the likelihoods for *t>1* of the full model (*p < 0.001*) are significantly higher than the next best model (dynamic). Accuracies for *t = 1* of the full model are greater than the next best model, the static model (*p = 0.07*), while the likelihoods for *t = 1* of the full model are not significantly greater than the likelihoods of the static model (*p = 0.31*). **Last four rows** are performance on average cooperation level in each structure (*n = 30*) and time series of average cooperation in each structure (*n = 212*). Infinitely repeated interactions with delta set to 0.5 are on average only two periods long and there is not sufficient empirical data to extend out to eight periods so we extend to seven. Two structures are finitely repeated for two periods and two others are finitely repeated for four periods. We conducted paired sample t-tests between the full model and competitors, with a null hypothesis that the true difference in means of the 212 squared errors between predicted and real cooperation levels at all times in all game structures is equal to zero, i.e. that the full model and a competitor are statistically indistinguishable in terms of squared errors on time series predictions. We did the same for the thirty predictions of overall cooperation levels. We reject the null of no difference for all comparisons except with the static model for both tests and the dynamic model for the time series (see S1 Appendix).

	Full	Static	Dynamic	fEWA	Baseline
**Acc. t = 1**	*68*	62	57	48	48
**Acc. t > 1**	*86*	68	85	62	62
**LL t = 1**	*-656*	-668	-846	-748	-761
**LL t > 1**	*-1624*	-2945	-1726	-3108	-3146
**Cor-Time**	*0.755*	0.709	0.713	0.241	-0.697
**Cor-Avg**.	0.774	*0.819*	0.721	0.106	-0.724
**RMSE-Time**	*0.149*	0.154	0.163	0.213	0.224
**RMSE-Avg**.	0.126	*0.113*	0.136	0.194	0.203

### Aggregate-level performance

**Fig 4:**
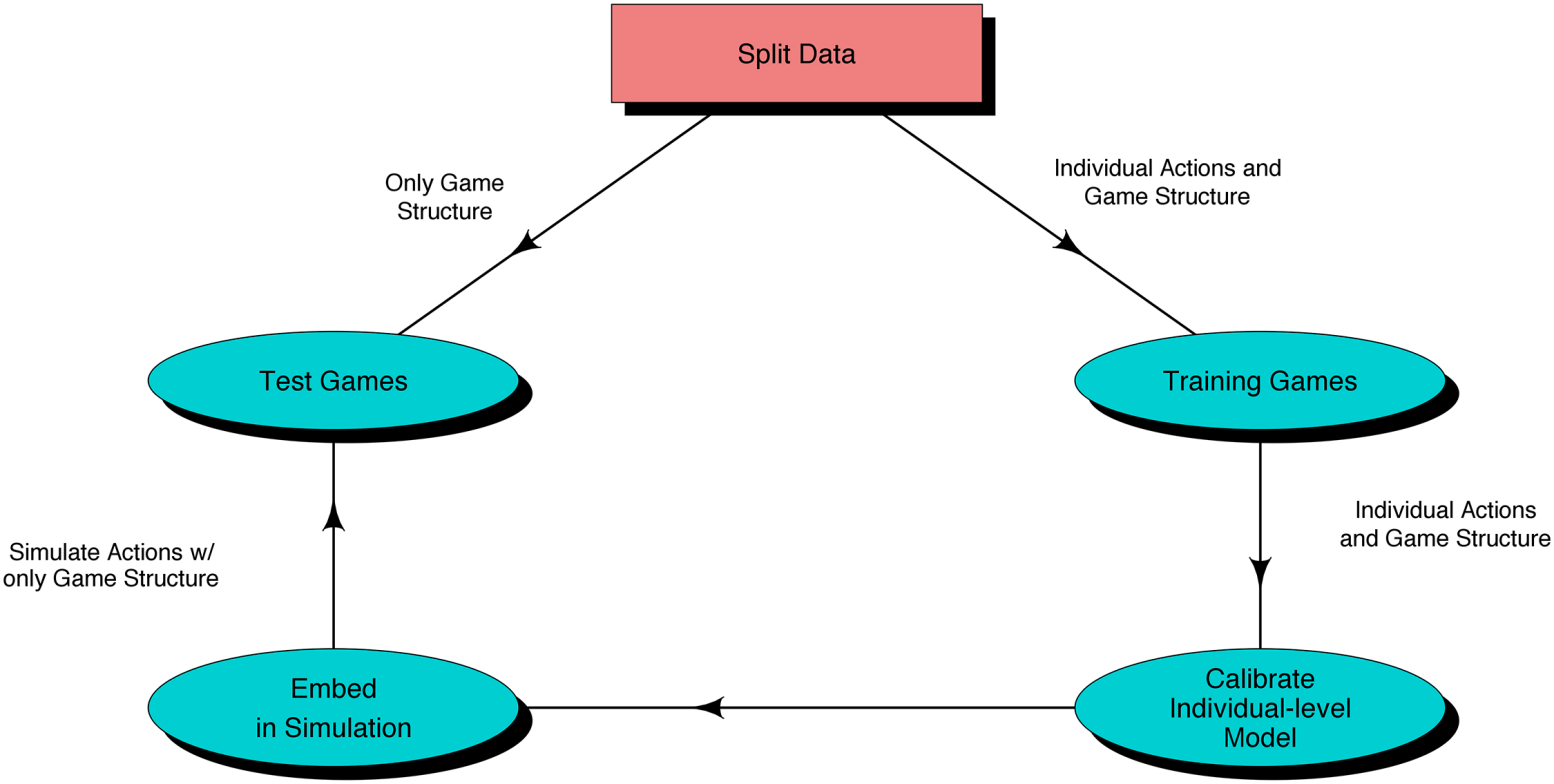
Model validation process for aggregate-level patterns. We tested the dynamic-only model by sampling lagged outcomes for ‘period zero’ actions from a Bernoulli distribution with equal probability of cooperation and defection, which is approximately the mean cooperation rate in the data. A subtle, but crucial, distinction between this process and the model validation process for individual-level action predictions (Fig 3) is that, here, *we only pass game structures* for the test games, rather than the full behavioral data and the game stucture.

To evaluate the model’s ability to predict behavior in new game structures, we developed the following procedure (Fig 4). Assign each of the thirty game structures into either training or test data. With the training data, learn the parameters of the individual-level model. Next, create a simulation in which the estimated individual-level model makes joint decisions in a repeated Prisoner’s Dilemma game, and predict the probabilistic behavior in game structures assigned to the test data *using only the game structure*, i.e., using no behavioral data from the experiment. Finally, compare the predictions, $p(y_{sim}^{new}|{\vec{Game}}^{new})$, to actual observed cooperation dynamics, $y_{obs}^{new}$, using both squared error and correlation to measure success of the model in predicting behavior. Repeat this process thirty times, including a different game structure as the held-out test each time (leave-out-one-cross-validation), until we have a prediction for each of the game structures as if each prediction were made before any data had been collected for that experimental design. We also test that the results are robust to the number of folds in the cross-validation procedure (from thirty down to two), i.e. robust to the number of game structures used for training.

We compare the performance of the five models’ predictions of average probability of cooperation and dynamics of cooperation (Table 3). Our model is slightly worse at predicting overall cooperation levels than the static model, but better at predicting dynamics (neither comparison is significant), and is significantly better than the other models in almost all cases.

**Fig 5:**
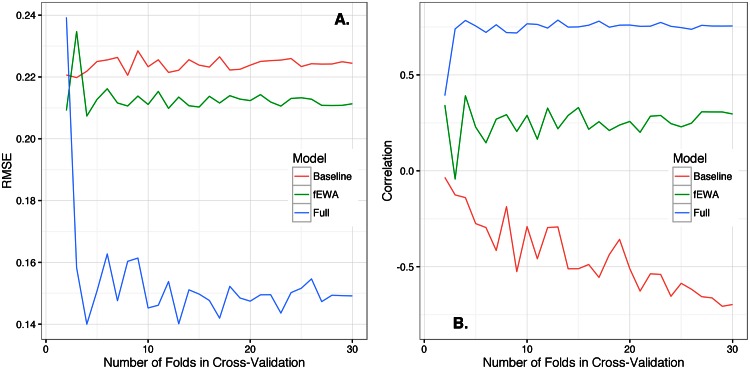
How predictive performance varies with data splitting. RMSE (**A**.) and correlation (**B**.) for time series forecasts of play in 30 game structures, varying folds in cross-validation from 30 to 2. The full model consistently has lower prediction error and higher correlation than the baseline model and the fEWA model until there are only two folds. It is, in general, difficult to make accurate predictions when the ratio of observational units to folds is small. In the case of predicting aggregate and dynamic play, the game structure itself is the observational unit, and we only have thirty, so it’s not surprising that performance can degrade at two folds depending on the particular random realization of fold assignments.

Estimating the parameters of the model on a subset of the data and then evaluating the performance of the model on held-out data allows us to measure generalizability. However, randomly dividing the data increases bias of the evaluation of the predictive performance because the estimated value of the predictive power is conditional on which data were included in the training or test samples. To reduce this bias, it is common to run multiple rounds of this process and then average the resulting values of predictive performance [41]. If we do this *n* times, this is called leave-out-one-cross-validation (LOOCV), which has lower bias; however, LOOCV can have higher variance in the estimates compared to *k*-fold validation, where *k* < *n* [42]. Fig 5 displays the effect of the number of folds in cross-validation on model performance, demonstrating that our main results are robust to the value of *k*.

**Fig 6:**
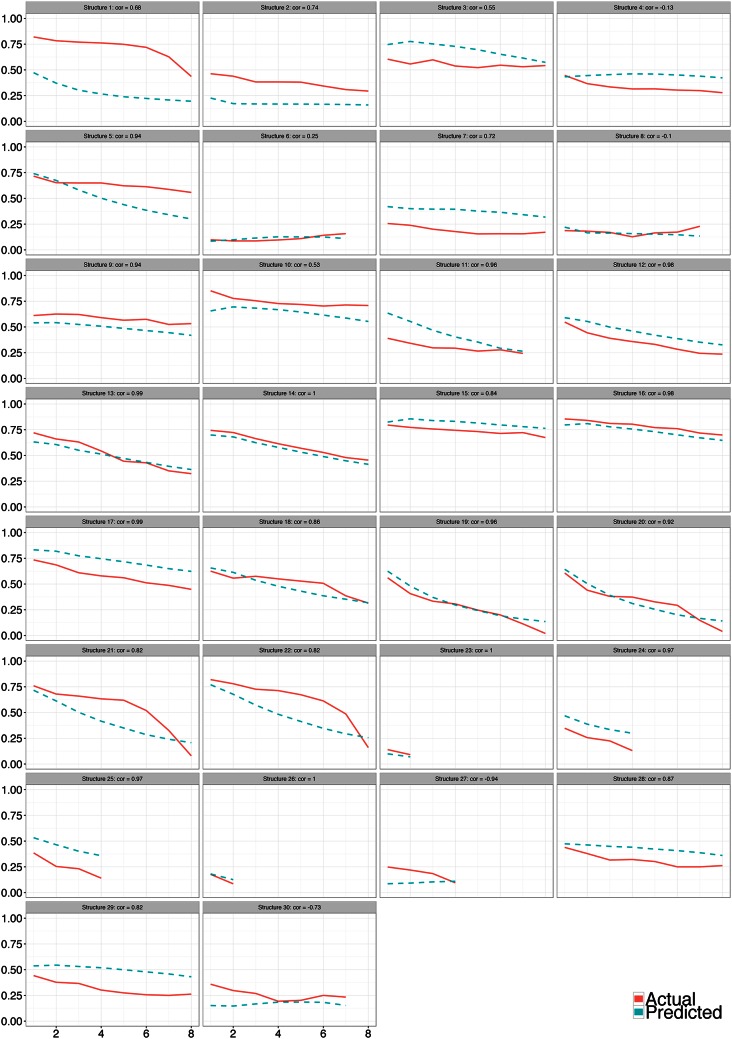
Model forecasts. Out-of-sample forecasts of cooperation level over time, for all game structures, conditional only on the game structure (*n* = 212).

Every panel in Fig 6 is the full model’s *out-of-sample forecast* for the average probability of cooperation at each time, conditional only on the game structure of that experiment. Our model’s time series of average cooperation is statistically significantly positively correlated (0.76, *p < 0.001*) with the observed time series. To better understand Fig 6, observe, for example, Structure 14: using no data from that game structure, our model predicted the initial (high) level of cooperation almost exactly and then was perfectly correlated with the empirically observed mean cooperation level throughout the next seven periods of play. The S1 Appendix displays the equivalent of Fig 6 for all other models, which are noticeably worse at predicting the time series.

**Fig 7:**
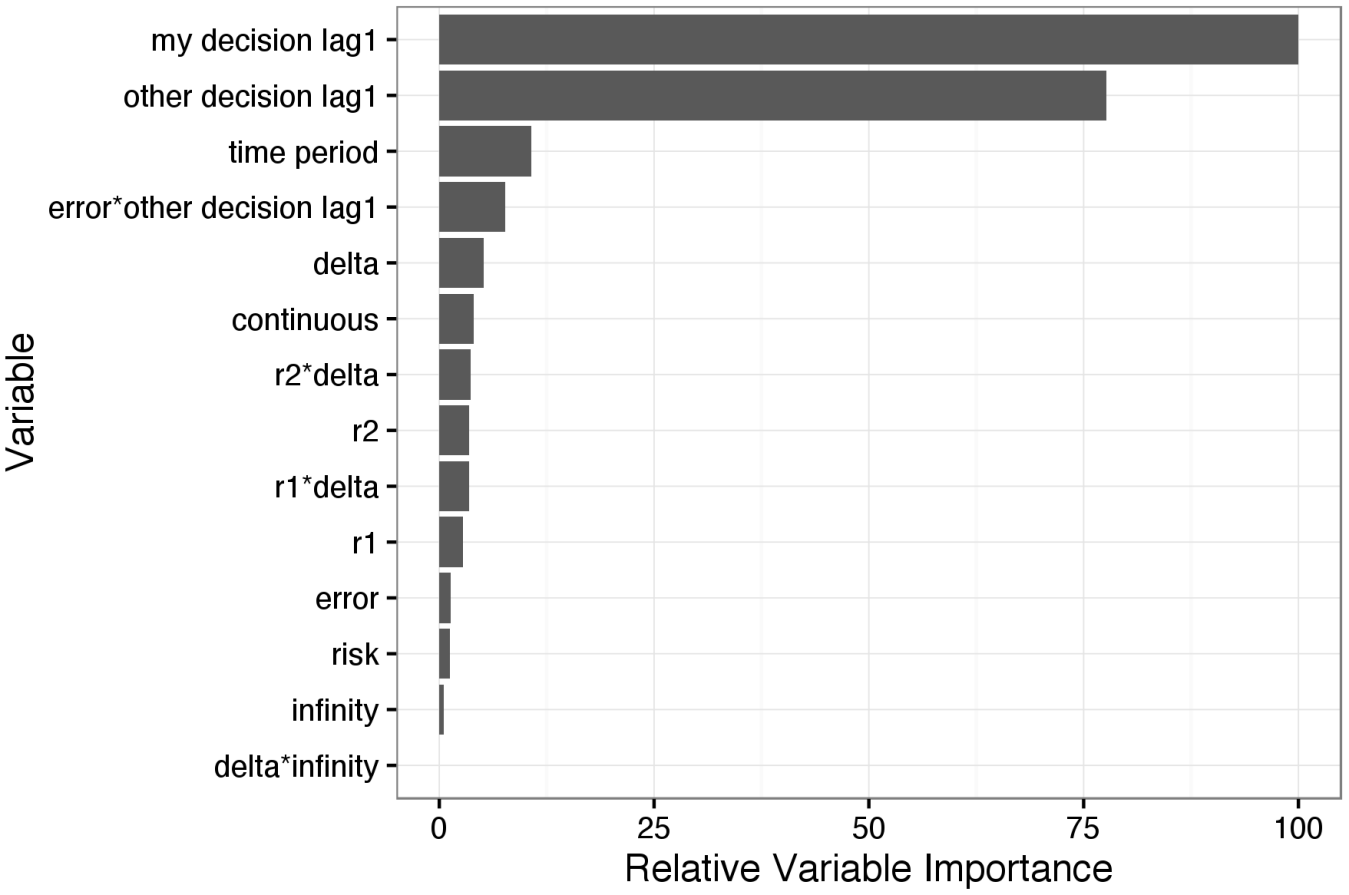
Variable importance scores. Variable importance scores for individual-level dynamic component of full model, i.e. for predictions of an agents’ probability of cooperation in periods *> 1*. Variables separated by ‘*’ represent an interaction between those two variables. These relative importance scores are derived from the absolute values of the t-statistics for each model parameter, which correspond to the effects of the predictor variables (accounting for variability in the estimates) on the probability of cooperation, *ceteris paribus* [19].

The dynamic-only model performed nearly as well as the full model on individual-level *period* > 1 actions, but worse on both tests of aggregate pattern predictions. By investigating the coefficients of the estimated individual-level dynamic model, we discover that the actions taken by a player and her opponent in the previous period are highly predictive of the next action (Fig 7). The variable with the most predictive power is the player’s own previous action: if a player cooperated (defected) in the previous period, she is very likely to cooperate (defect) in the next. There is strong inertia to Prisoner Dilemma behavior, and, therefore, accurate prediction of first period play is crucial for good performance at the aggregate level. fEWA can incorporate the payoff game structure variables but not the other variables, which prevents high first period accuracy. The full model is able to predict first period play well with a model trained only on first periods in the training data, and then use a dynamic model trained on periods >1 in the training data, allowing for subtly different relationships between game structures and the evolution of cooperation.

**Fig 8:**
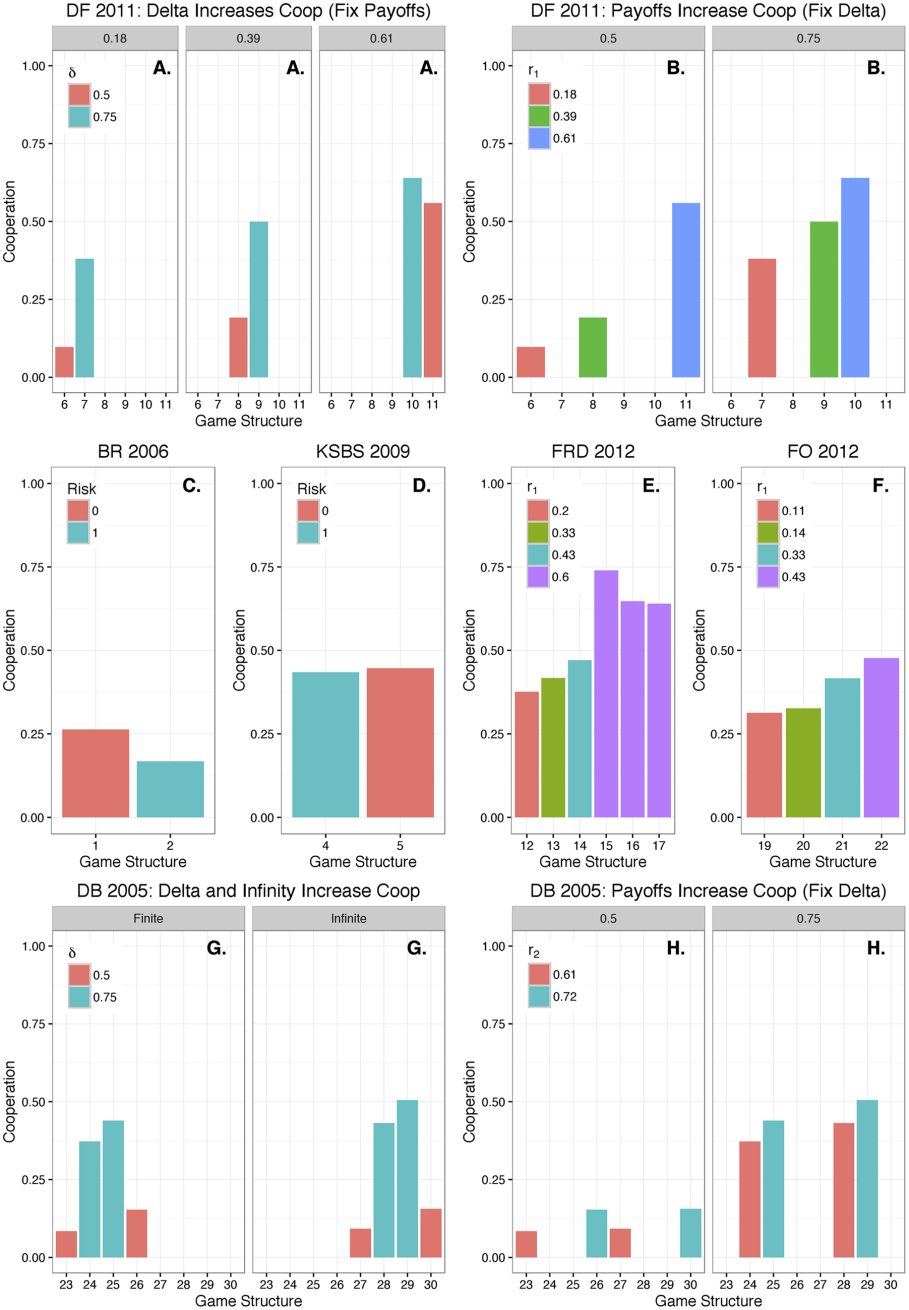
Predicted proportions of cooperation (*n = 28*). We could not include more than one game structure from two papers (game structures 3 and 18) that comprised our integrated data set [6, 9], because they were comparing either to one-shot games or games with artificial opponents. Therefore, in our model’s replication of the qualitative empirical experimental findings, we could not conduct any replication related to these two papers’ findings. Dal Bo and Frechette found that: delta increases cooperation, keeping payoffs fixed (**A**.); and that certain payoffs increase cooperation, while fixing delta (**B**.). Bereby-Meyer and Roth found that risk reduces cooperation, where payoffs were framed as gains (**C**.). Kunreuther et al. found that risk reduces cooperation, with payoffs framed as losses rather than gains (**D**.). This is the only finding where we predicted marginally different cooperation levels when the empirical data indicates a larger gap. Fudenberg Rand and Dreber found that certain payoffs increase cooperation (**E**.). Friedman and Oprea found that certain payoffs increase cooperation (**F**.). Dal Bo found that delta increases cooperation, fixing payoffs and infinity (**G**.); having an ‘infinitely’ repeated game increases cooperation, fixing payoffs and delta (**G**.); and certain payoffs increase cooperation, fixing infinity and delta (**H**.). Dal Bo also found that the cooperation levels decrease more over time within finite games (Fig 6 Structures 23–26), compared to infinite games (Fig 6 Structures 27–30).

The empirical experiments varied structural game parameters to measure hypothesized differences in cooperation levels between structures. As a final validation, we compared the (out-of-sample) predicted average cooperation levels between our synthetic model of behavior to the actual observed behavior in experiments [7, 8, 10–13]. Overall, our model came to the same qualitative conclusions as the experiments: *δ*, infinity and particular payoff configurations increased cooperation, while risk reduced cooperation. We detail each paper’s finding and illustrate our model’s corresponding finding graphically in Fig 8.

## Analysis

After re-learning the computational model with all available data to best explore the full parameter space, we deployed it to quantify the sensitivity of cooperation to each of the structural game design parameters. To systematically explore the model, we generated thousands of collections of input values (specifications of Prisoner’s Dilemma experiments) from the multi-dimensional distribution covering the feasible ranges of all input values using Latin Hypercube sampling [43, 44]. The variables are drawn from the following distributions, with the constraint that *r*_1_ < *r*_2_ because *r*_1_ is always less than *r*_2_ in the data: *error* ∼ *Unif*(0,0.5); *δ* ∼ *Unif*(0.45,0.95); *infinity* ∼ *Bern*(0.5); *risk* ∼ *Bern*(0.5); *r*_1_ ∼ *Unif*(0,1); *r*_2_ ∼ *Unif*(0,1). Then we simulated cooperation dynamics for each experimental input set. This global sampling and simulation allows subsequent analysis to generate reliable information about the relationships between model inputs (structural game design parameters) and output (cooperation behavior) [45, 46].

**Fig 9:**
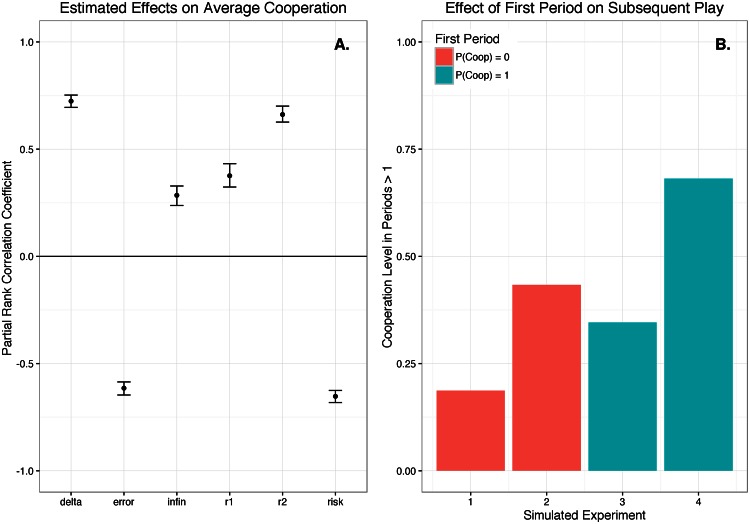
Model simulation analysis. **A**. is a partial rank correlation coefficient analysis [47] of the effects of the game parameters on average cooperation; lines are bootstrapped 95% confidence intervals (*n = 1,000*). Continuous is set to its empirical mode, 0, because we had no within experiment variation on this. **B**. shows that first period play strongly affects cooperation levels in periods greater than one. Setting the game structure variables to the mean of the empirically observed values: if we exogenously set the probability of cooperation during the first period to 0 the simulated proportion of cooperation in subsequent periods is only 0.18 (‘simulated experiment 1’), and if we set the probability of cooperation during the first period to 1 the simulated proportion of cooperation in subsequent periods is 0.68 (‘simulated experiment 4’). When the probability of first period cooperation is set to 0, and we use a game structure that **A**. suggests should maximize cooperation, the proportion of cooperation is 0.43 (‘simulated experiment 2’); and when the probability of first period cooperation is set to 1, with the game structure that should minimize cooperation, the cooperation level is 0.35 (‘simulated experiment 3’).

Based on the results of a partial rank correlation coefficient analysis [45, 47], the six main game structure variables can be divided into three groups that contain two variables each within the 95% confidence interval of each other (Fig 9A); we obtain qualitatively equivalent results with a standardized rank regression coefficient analysis (see S1 Appendix). *δ* and *r*_2_ have *very large positive effects* on average cooperation levels. As noted above and explained further in the S1 Appendix, our *δ* measure is applicable to both infinite and finite games as a measure of the expected length of the game from a first period perspective, and the dynamic model has an interaction term between *δ* and *infinity* that allows the *δ* effect in periods greater than one to be different for infinite games. Surprisingly, this interaction term is the least important predictor variable in the dynamic model (Fig 7), suggesting that the effect of the expected length of the game from a first period perspective is independent of whether the game is indefinitely repeated.

*Infinity* and *r*_1_ have *moderately large positive effects* on cooperation. *r*_1_ is generally used as an index of the cooperativeness of the payoff table so it is surprising that *r*_2_ has a significantly larger impact on cooperation. Our analysis suggests that we can increase the probability of cooperation more by increasing the difference between the potential outcomes of a player and her opponent both cooperating (C,C) and only her cooperating (C,D). Increasing the difference between mutual cooperation (C,C) and mutual defection (D,D) will also increase cooperation, but less. The third group includes *error* and *risk*, which have *negative effects* on cooperation.

We empirically discovered that if a player cooperated (defected) in the previous period, she is very likely to cooperate (defect) in the next (Fig 7). To explore the implications of this finding, we modified our simulation model so that we could exogenously set the probability of an agent cooperating in the first period, and found that it strongly affects cooperation levels in subsequent periods with the game structure set to the empirical mean values (Fig 9B Simulated Experiments 1 and 4). However, a game structure that the sensitivity analysis indicates is very favorable to cooperation can moderate the negative effect of initial defection (Fig 9B Experiment 2), and, conversely, a game structure that the analysis suggests should inhibit cooperation can moderate the positive effect of initial cooperation (Fig 9B Experiment 3). The history of a particular interaction *and* the institutional structure both play important roles in determining cooperation levels.

We further investigated “inertia”—the probability a player will cooperate given that she cooperated last period—and its relationship to game structure. We compute an average “predicted inertia” for each of the thirty game structures by predicting the probability of cooperation after cooperating last period in a given game structure with our model, marginalizing out the effect of the time period and the opponent’s previous decision. To compute an average “actual inertia” value for each of the thirty game structures we divide the sum of the number of times all players cooperated after cooperating in the previous period by the total number of times all players cooperated in the previous period. The thirty predicted and actual inertia values have a 0.74 correlation, further evidence that the model captures the relevant patterns in the data. Game structures with longer expected length of interactions from a first-period perspective (higher *δ*), indefinite repetition, and higher *r*_2_ payoff values have higher actual inertia values. *δ* is the strongest predictor of higher inertia and they are correlated at the 0.71 level.

## Conclusion

The Prisoner’s Dilemma game is widely used to understand the tension between social and individual interests. We develop a computational model that can accurately predict human behavior in Prisoner’s Dilemma experimental games for a broad range of game structures, using only separate such structures for calibrating the model. We demonstrate that our approach can successfully predict behavior at multiple scales, yielding the most rigorously and broadly validated computational framework to date for designing institutions that promote cooperation in social dilemma scenarios. In particular, we use our model to identify variables that have the greatest impact on cooperation.

Our sensitivity analysis demonstrated the importance of higher expected values of interaction length and larger differences between potential *C,C* and *C,D* outcomes (Fig 9). It is more important to increase the benefits of mutual cooperation over losing out by being the sole cooperator than it is to increase the potential benefits of mutual cooperation relative to mutual defection. These insights are relevant to improving the underlying structure of new policy programs and designing new human subjects experiments. This work represents a new approach to understanding and *predicting* human interactions that will be increasingly relevant as more (experimental and observational) behavioral data is collected. With sufficient behavioral data from a variety of policy structures, our approach can be applied to understand which factors should be prioritized to improve policy outcomes. The specifics need to be tailored to the circumstance, but models like ours can serve as a starting point for understanding which structural factors of a policy are most influential.

## Supporting Information

S1 Appendix Supplementary Information. (PDF)

S2 Appendix Dataset. (ZIP)
