# Supplementary material for: Predicting Human Cooperation
Source: PLoS One. 2016 May 12;11(5):e0155656. doi: 10.1371/journal.pone.0155656 (PMC4865178; doi:10.1371/journal.pone.0155656)
Supplement: S1 Appendix — (PDF) [file pone.0155656.s001.pdf]

Supplementary Information for: Nay and Vorobeychik "Predicting Human Cooperation"

John J. Nay, Yevgeniy Vorobeychik

Feb 8, 2016

What are the details of the data sets?

For the fEWA model (see details of this model below), which uses the raw payoffs, we converted payoffs to the equivalent amount in U.S. dollars that the participant in the experiment would have received: DF 1 payoff = \$0.006; DO 1 payoff = \$0.01; FR 1 payoff = \$0.033; KS 1 payoff = \$0.10; BR 1 payoff = \$1.0; AM 1 payoff = \$1.0; and FO 1 payoff = \$0.006 ($\frac{\$0.05}{8}$).

Fig. S1 displays the distribution of the values of the time period in our full combined data-set at the individual action level (n = *168,386*) and the distribution of the proportion of cooperation in our full combined data-set at the game structure level (n = *30*).

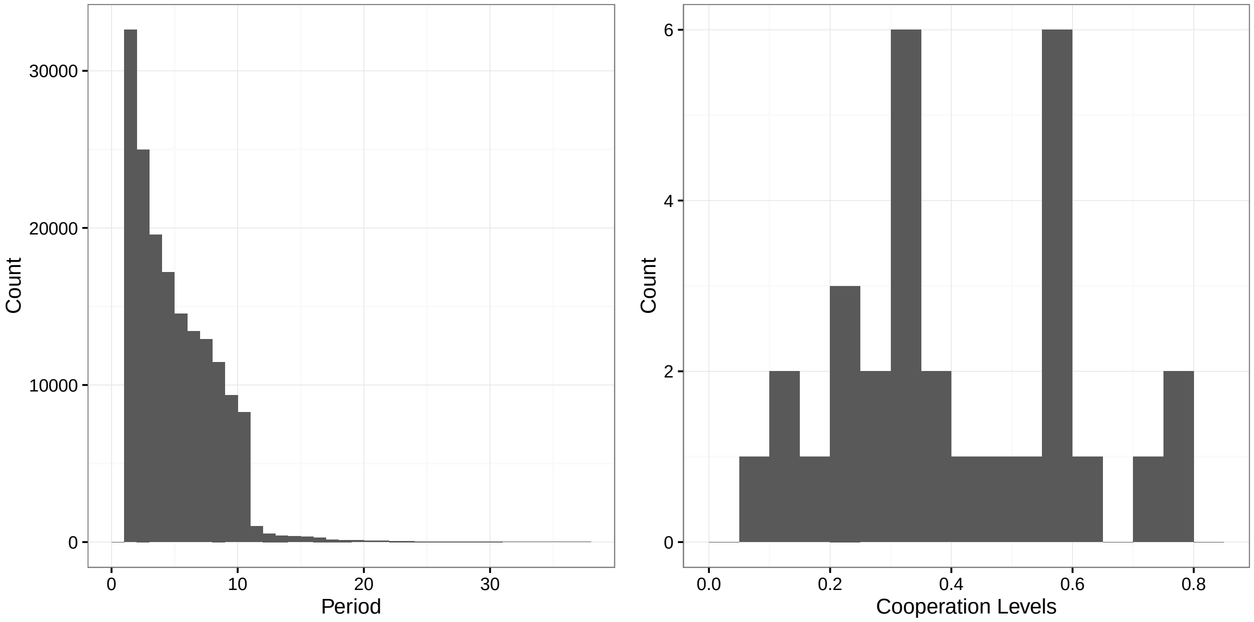

Fig. S1. Left is the empirical density of time periods for all individual-level action observations in data. Right is the empirical density of the cooperation proportions at the game structure level.

What are the details of the fEWA Model?

This section is summarizing parts of [1,2]; all the equations are directly from [1,2]. We summarize this information from those papers here for our readers to easily access the details of the model.

Camerer and Ho [3] created the experience-weighted attraction (EWA) model as a hybrid of reinforcement and belief models, the two primary behavioral game theory models that preceded it.

For player i, there are m (two for the Prisoner's Dilemma) strategies indexed by j.

- Each player has an initial attraction for each strategy, $A_i^j(0)$.

- i's chosen strategy is $s_i(t)$.
- i's opponent's chosen strategy is $s_{-i}(t)$.
- i's payoffs are $\pi_i(s_i^j(t), s_{-i}(t))$.
- The indicator function yields $I(x,y) = 0$ if $x \neq y$, and $I(x,y) = 1$ if $x = y$.

Attractions, $A_i^j(t)$, are updated after each time period, t, for each strategy, j, for each player, i, with following equation.

$$A_i^j(t) = \frac{\phi N(t-1)A_i^j(t-1) + [\delta + (1-\delta)I(s_i^j, s_i(t))]\pi_i(s_i^j, s_{-i}(t))}{N(t-1)\phi(1-\kappa)+1}$$

Then, attractions are mapped into probabilities of choosing discrete actions during the next time period (Cooperate and Defect for the Prisoner's Dilemma) with a logistic stochastic response function, which has a parameter, λ, that controls how responsive the agent is to differences in attractions. If $\lambda = 0$, the choice is made randomly and is independent of the attraction values.

$$P_i^j(t+1) = \frac{e^{\lambda A_i^j(t)}}{\sum_{k=1}^{m_i} e^{\lambda A_i^k(t)}}$$

In order to address overfitting EWA to data, Ho, Camerer and Chong [1] created a one parameter version of EWA, functional (self-tuning) experience-weighted attraction learning (fEWA), that is designed to forecast behavior in new games where there is no data from that game to estimate parameters. Thus, this model is the state-of-the-art behavioral game theoretic model for our purposes.

fEWA replaces the free parameters of EWA with functions of the data that can vary across players and time. Following Camerer and Ho [2], we set κ to 0 because this captures almost all of the familiar cases of estimation of EWA on a variety of data sets from different games and different populations. Then the other two parameters of EWA, ϕ and δ, are replaced by functions of player i's experience up to period t: $\phi_i(t)$ and $\delta_{ij}(t)$.

$\phi_i(t)$ captures how quickly a player perceives the environment to be changing and if there is more change less weight is placed on attractions. This is accomplished by creating a "surprise index" of the degree of change of the most recent observation of opponent's play from the interaction history. The surprise index is a function of two vectors. A player has a *cumulative history vector* of the other player's choice of strategy k at each period up until the current period. An element of this vector is $h_i^k(t) = \frac{\sum_{\tau=1}^{t} I(s_{-i}^k, s_{-i}(\tau))}{t}$. The player also creates an *immediate history vector* of 0's and 1's, where there is a 1 for the strategy played that period $s_{-i}^k = s_{-i}(t)$ and a 0 for the other strategy. An element of this vector is $r_i^k(t) = I(s_{-i}^k, s_{-i}(t))$. The surprise index at each time is then $S_i(t) = \sum_{k=1}^{m_{-i}} (h_i^k(t) - r_i^k(t))^2$. $S_i(t) \in (0,2)$, and $\phi_i(t) = 1 - \frac{1}{2}S_i(t)$. $\phi_i(t) \in (0,1)$.

The attention function, $\delta_{ij}(t)$, is the weight placed on forgone payoffs. Players reinforce chosen strategies and unchosen strategies with profits *larger* than the profits in the current period by a weight of one, and reinforce unchosen strategies with profits *smaller* than the profits in the current period by a weight of zero. If $\pi_i(s_i^j, s_{-i}(t)) \geq \pi_i(t)$, then $\delta_{ij}(t) = 1$. If $\pi_i(s_i^j, s_{-i}(t)) < \pi_i(t)$, then $\delta_{ij}(t) = 0$.

Therefore, the attractions are updated by the following equation, where $\phi_i(t)$ and $\delta_{ij}(t)$ are defined as above.

$$A_i^j(t) = \frac{\phi_i(t)N(t-1)A_i^j(t-1) + [\delta_{ij}(t) + (1-\delta_{ij}(t))I(s_i^j, s_i(t))]\pi_i(s_i^j, s_{-i}(t))}{N(t-1)\phi_i(t)+1}$$

Like our logistic regression models, our computational simulation model implementation uses *probabilities* of actions to predict average cooperation levels. We use the logistic stochastic response function, $P_i^j(t+1) = \frac{e^{\lambda A_i^j(t)}}{\sum_{k=1}^{m_i} e^{\lambda A_i^k(t)}}$, to map fEWA attractions into the probability of cooperation. This is the standard choice model used for fEWA and other behavioral game theories [2]. We estimate the λ parameter of the response function

by searching for a value that minimizes the negative log likelihood of the model on the training data. We used the base R stats::optimize implementation of a combination of golden section and successive parabolic interpolation to search for the optimal values.

We derived initial attractions, $A_i^j(0)$ by simulating period zero play as random choices, the same way we imputed lagged decisions for period one play for the dynamic-only logistic regression model. The initial experience weight, $N(-1)$, is set to 1, and at period zero, we randomly draw both players' actions from a Bernoulli distribution with equal probability over the two possible actions, then we update attractions based on those actions and the associated simulated payoffs assuming equal initial attraction, $A_i^j(-1)$:

$$A_i^j(0) = \frac{[\delta_{ij}(0) + (1 - \delta_{ij}(0))I(s_i^j, s_i(0))]\pi_i(s_i^j, s_{-i}(0))}{N(-1)\phi_i(0) + 1}$$

Then, as play progresses in an interaction, attractions are updated according to the EWA formula. Because fEWA uses the raw payoff values to determine attractions to actions, we converted all payoff values in the data to dollars and then for each game structure we normalized payoff values by subtracting each by the lowest possible payoff for that game structure, which is important to help the model cross-game forecast [1].

Why a global function for all players, rather than heterogeneous agents?

Studies of individual behavior, such as laboratory games, often conduct multiple-type and even individual-level estimation, where human participants are clustered into segments and then the behavioral models' parameters are estimated within those segments. In the case of individual-level estimation, the segments are individual players, and so the models' parameters are estimated for each individual player; see [4] for an application of this approach to public goods games. In laboratory games, a human participant's "type" can be estimated using their observed actions. It can be more effective to incorporate observed actions as features in a model that is learned by directly predicting actions than to use unsupervised clustering as a preprocessing step. With our approach, agents can be heterogeneous by having different values of the features used in the behavioral model.

Our model adds a touch of randomness to each agent's observations of the two actions taken in the previous period of the current interaction to simulate heterogeneity. Fig. S2 shows a million draws from the normal distribution, N($\mu = 0$, $\sigma = 0.3$), that was used to draw a value to add to the probability of cooperation of each agent in the previous period at each time period > 1.

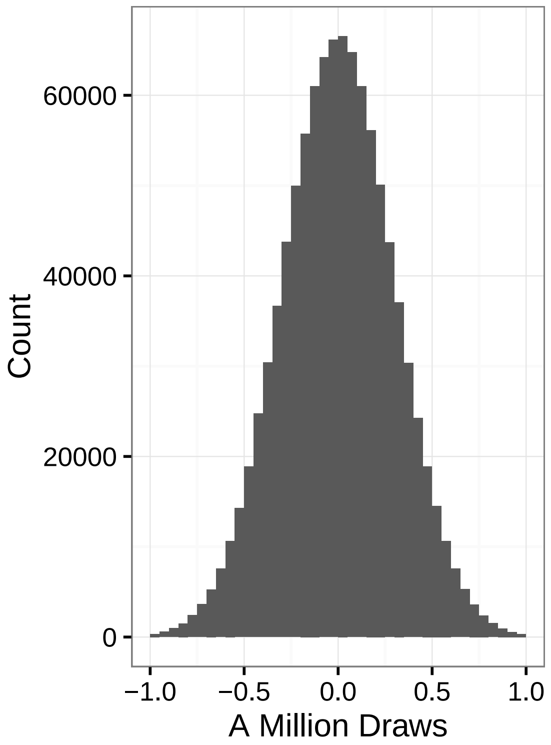

Fig. S2. Draws from the normal distribution that our computational model uses to add randomness to past action probabilities.

What are the details of the model comparisons?

Table S1 lists the p-values for the aggregate-level model comparisons.

	Static	Dynamic	fEWA	Baseline
**Time Errors**	0.485	*0.004*	*< 0.001*	*< 0.001*
**Avg. Errors**	0.279	0.367	*0.017*	*0.006*

Table S1. P-values for paired sample t-tests (not assuming equal variances) of differences between time errors (test three) and average cooperation level errors (test two) of full model and the corresponding errors of competing models: $p > 0.05$ suggests that the model has similar performance to the full model. We reject the null of no difference for all comparisons except with the static model for both tests and the dynamic model for the time series.

Time series plots for all models

Fig. S3 displays predictions of dynamic cooperation with the static-only model, Fig. S4 displays predictions of dynamic cooperation with the dynamic-only model, Fig. S5 displays predictions of dynamic cooperation with the null model, and Fig. S6 displays predictions of dynamic cooperation with the fEWA model.

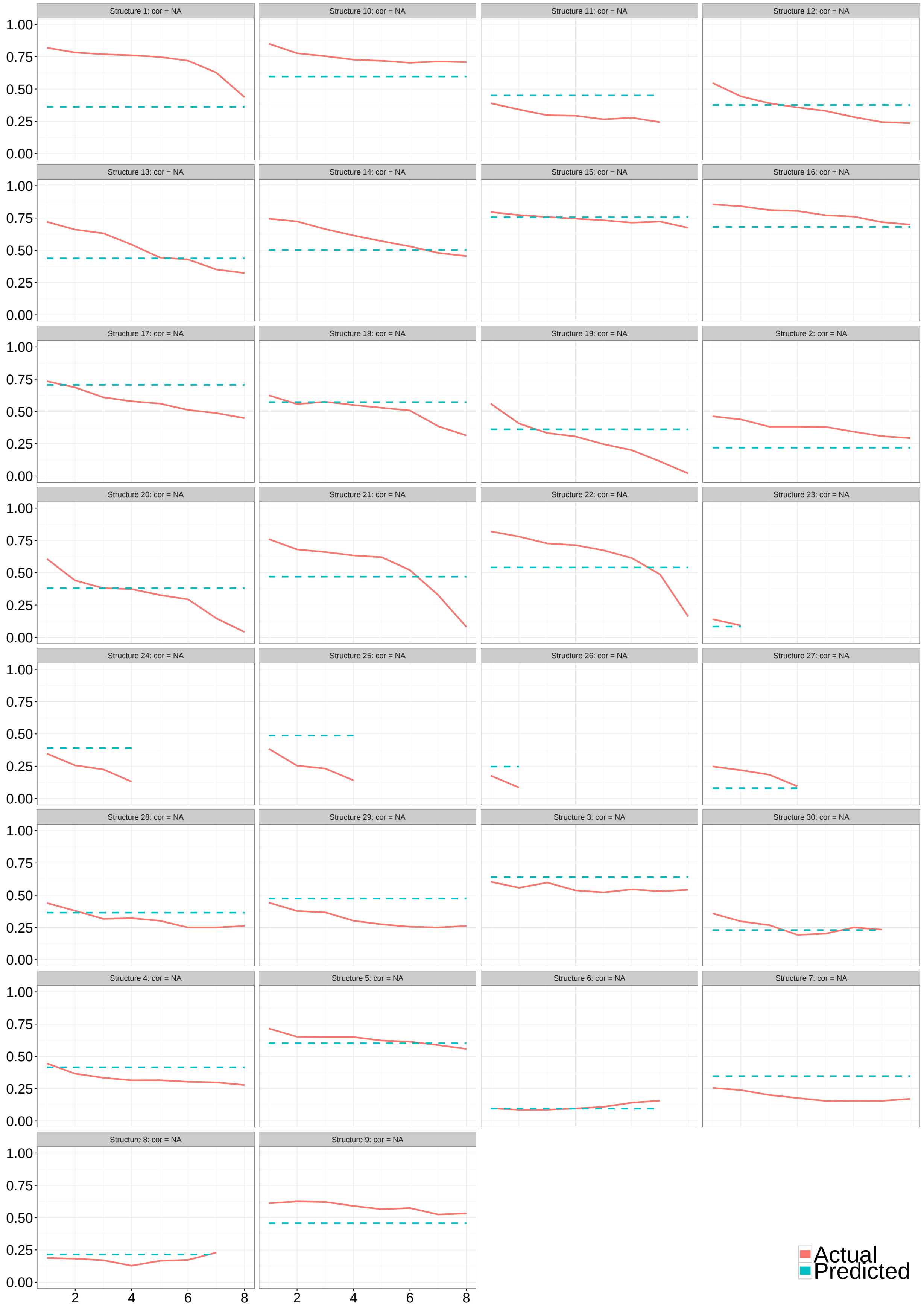
Structure 1: cor = NA
Structure 10: cor = NA
Structure 11: cor = NA
Structure 12: cor = NA
Structure 13: cor = NA
Structure 14: cor = NA
Structure 15: cor = NA
Structure 16: cor = NA
Structure 17: cor = NA
Structure 18: cor = NA
Structure 19: cor = NA
Structure 2: cor = NA
Structure 20: cor = NA
Structure 21: cor = NA
Structure 22: cor = NA
Structure 23: cor = NA
Structure 24: cor = NA
Structure 25: cor = NA
Structure 26: cor = NA
Structure 27: cor = NA
Structure 28: cor = NA
Structure 29: cor = NA
Structure 3: cor = NA
Structure 30: cor = NA
Structure 4: cor = NA
Structure 5: cor = NA
Structure 6: cor = NA
Structure 7: cor = NA
Structure 8: cor = NA
Structure 9: cor = NA
0.00
0.25
0.50
0.75
1.00
2
4
6
8
Actual
Predicted

Fig. S3. Predictions of dynamic cooperation with only static model.

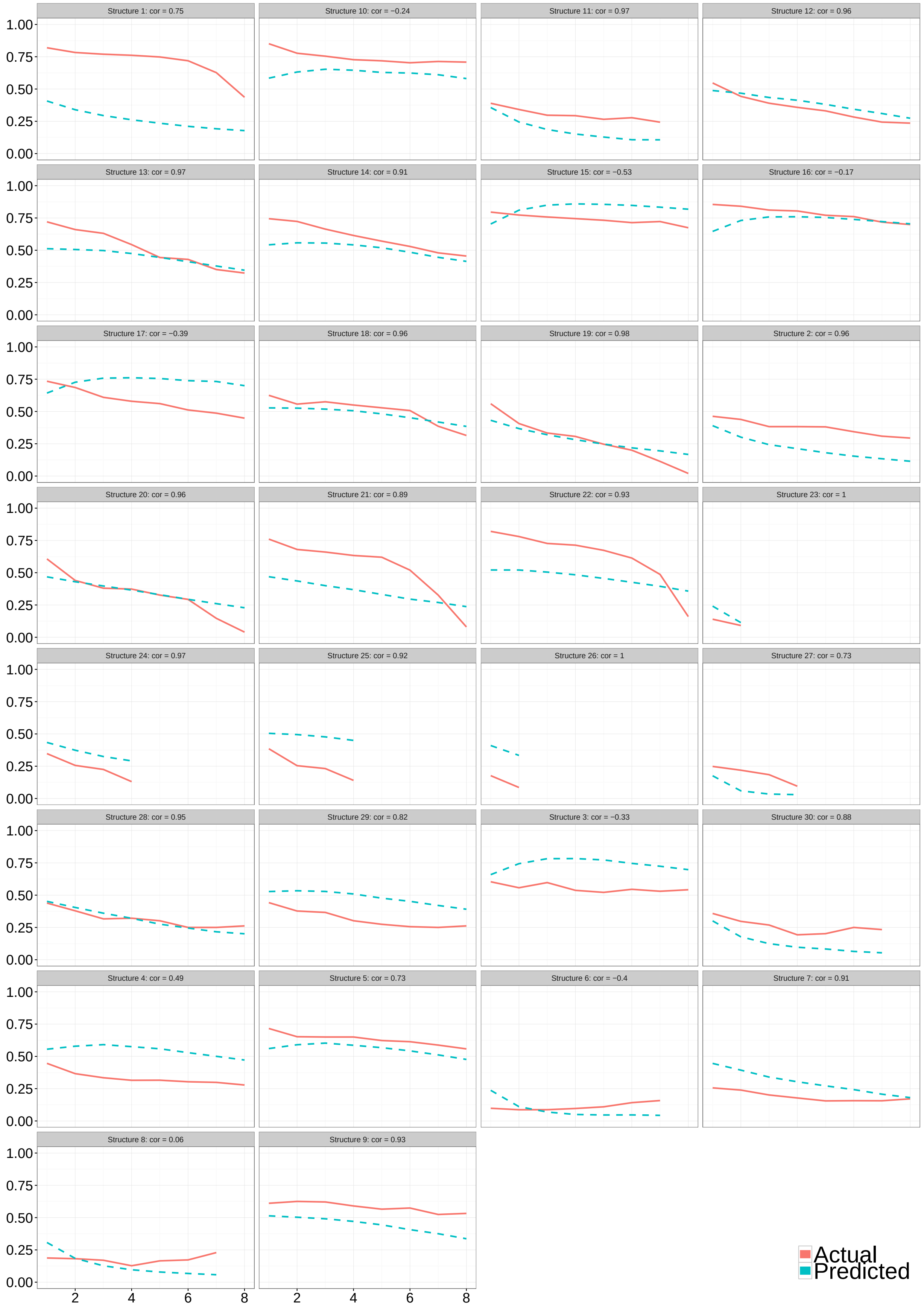

Structure 1: cor = 0.75
Structure 10: cor = −0.24
Structure 11: cor = 0.97
Structure 12: cor = 0.96
Structure 13: cor = 0.97
Structure 14: cor = 0.91
Structure 15: cor = −0.53
Structure 16: cor = −0.17
Structure 17: cor = −0.39
Structure 18: cor = 0.96
Structure 19: cor = 0.98
Structure 2: cor = 0.96
Structure 20: cor = 0.96
Structure 21: cor = 0.89
Structure 22: cor = 0.93
Structure 23: cor = 1
Structure 24: cor = 0.97
Structure 25: cor = 0.92
Structure 26: cor = 1
Structure 27: cor = 0.73
Structure 28: cor = 0.95
Structure 29: cor = 0.82
Structure 3: cor = −0.33
Structure 30: cor = 0.88
Structure 4: cor = 0.49
Structure 5: cor = 0.73
Structure 6: cor = −0.4
Structure 7: cor = 0.91
Structure 8: cor = 0.06
Structure 9: cor = 0.93
1.00
0.75
0.50
0.25
0.00
2
4
6
8
Actual
Predicted

Fig. S4. Predictions of dynamic cooperation with only dynamic model.

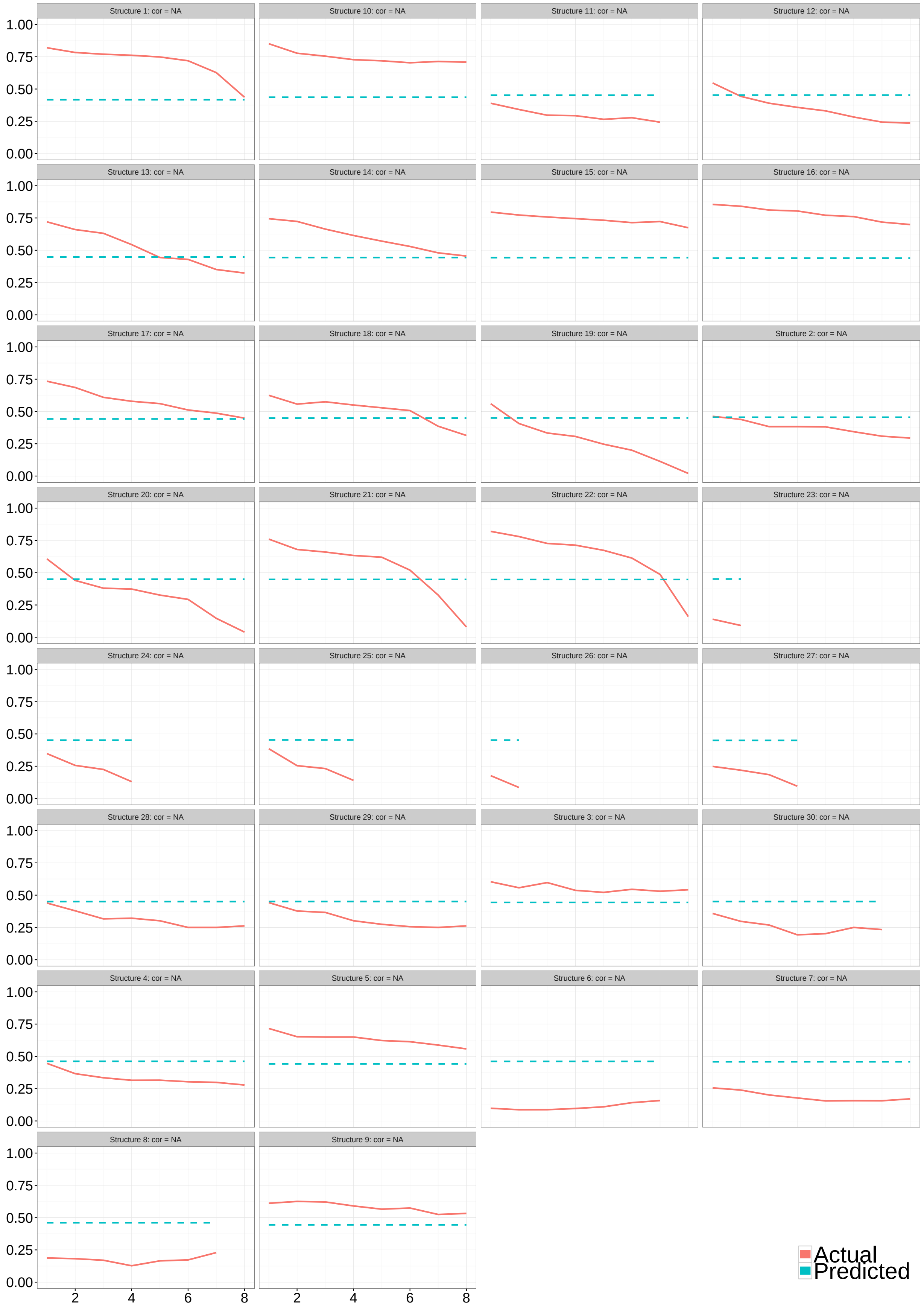
Structure 1: cor = NA
Structure 10: cor = NA
Structure 11: cor = NA
Structure 12: cor = NA
Structure 13: cor = NA
Structure 14: cor = NA
Structure 15: cor = NA
Structure 16: cor = NA
Structure 17: cor = NA
Structure 18: cor = NA
Structure 19: cor = NA
Structure 2: cor = NA
Structure 20: cor = NA
Structure 21: cor = NA
Structure 22: cor = NA
Structure 23: cor = NA
Structure 24: cor = NA
Structure 25: cor = NA
Structure 26: cor = NA
Structure 27: cor = NA
Structure 28: cor = NA
Structure 29: cor = NA
Structure 3: cor = NA
Structure 30: cor = NA
Structure 4: cor = NA
Structure 5: cor = NA
Structure 6: cor = NA
Structure 7: cor = NA
Structure 8: cor = NA
Structure 9: cor = NA
1.00
0.75
0.50
0.25
0.00
2
4
6
8
Actual
Predicted

Fig. S5. Predictions of dynamic cooperation with null model.

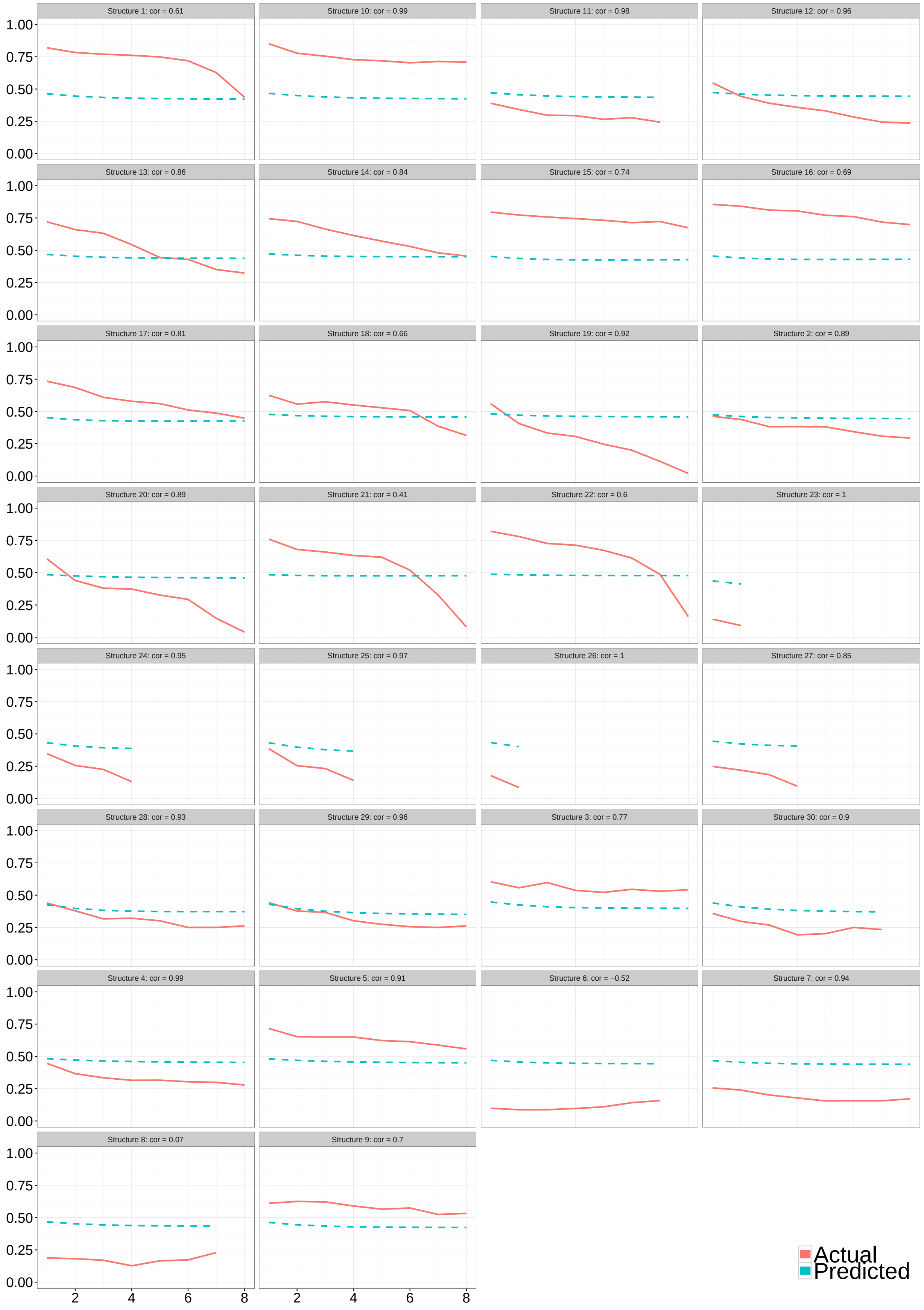

Structure 1: cor = 0.61
Structure 10: cor = 0.99
Structure 11: cor = 0.98
Structure 12: cor = 0.96
Structure 13: cor = 0.86
Structure 14: cor = 0.84
Structure 15: cor = 0.74
Structure 16: cor = 0.69
Structure 17: cor = 0.81
Structure 18: cor = 0.66
Structure 19: cor = 0.92
Structure 2: cor = 0.89
Structure 20: cor = 0.89
Structure 21: cor = 0.41
Structure 22: cor = 0.6
Structure 23: cor = 1
Structure 24: cor = 0.95
Structure 25: cor = 0.97
Structure 26: cor = 1
Structure 27: cor = 0.85
Structure 28: cor = 0.93
Structure 29: cor = 0.96
Structure 3: cor = 0.77
Structure 30: cor = 0.9
Structure 4: cor = 0.99
Structure 5: cor = 0.91
Structure 6: cor = −0.52
Structure 7: cor = 0.94
Structure 8: cor = 0.07
Structure 9: cor = 0.7
1.00
0.75
0.50
0.25
0.00
2
4
6
8
Actual
Predicted

Fig. S6. Predictions of dynamic cooperation with fEWA model.

What are the details of the sensitivity analysis?

Simulation models are used to investigate modeled systems by varying inputs to the model and measuring the effect on outputs. Input values are often varied one at a time, while keeping the others at "default values," but this prohibits the model analyst from discovering potential interaction effects and provides sensitivities that are conditional on the default values used [5,6]. To mitigate these issues, we base our sensitivity analysis on a global Latin Hypercube Sampling of the game structure variables [6–8]. We then analyze the relationship between the matrix of Latin Hypercube Sampled input parameters and the resulting vector of simulated outputs with two statistical methods. A partial correlation coefficient analysis estimates the linear effect of increasing or decreasing a given input on the output value, linearly discounting the effects of the other inputs. We estimate the partial *rank* correlation coefficient for each game design variable, which can capture non-linear (but monotonic) relationships [6,9,10].

We also conducted a standardized rank regression coefficient analysis to confirm that our results are robust to the specific analysis technique [9,10]. Furthermore, a standardized rank regression coefficient analysis can provide an R^2 value estimating the amount of variation explained [6]. The standardized rank regression coefficient analysis ranks the variables the same as the partial rank correlation coefficient analysis (Fig. S7), and has an $R^2 = 0.83$, which indicates that most of the variation in the simulation model is explained by the analysis.

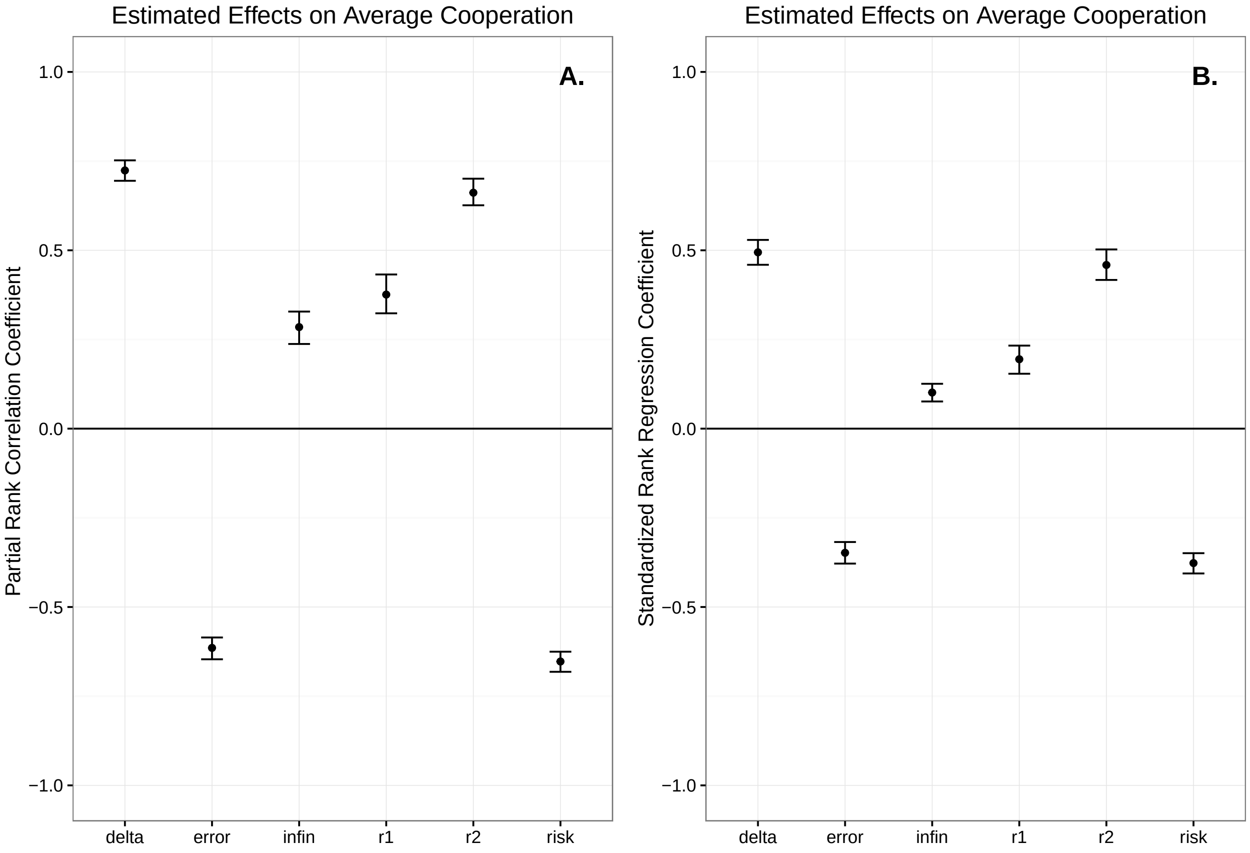

Fig. S7. Sensitivity analyses based on a Latin Hypercube Sampling design. The two techniques deliver the same qualitative results. **A.** The partial rank correlation coefficient sensitivity analysis also displayed in the main text. **B.** The standardized rank regression coefficient sensitivity analysis.

Software Information

R version 3.2.3 (2015-12-10) Platform: x86_64-apple-darwin15.2.0 (64-bit) Running under: OS X 10.11.3 (El Capitan)

attached base packages: [1] grid stats graphics grDevices utils datasets methods
[8] base

other attached packages: [1] eat_0.0.1 gtools_3.5.0 Hmisc_3.17-1 Formula_1.2-1
[5] survival_2.38-3 caret_6.0-64 lattice_0.20-33 pander_0.6.0
[9] ggplot2_2.0.0 knitr_1.12.3

loaded via a namespace (and not attached): [1] Rcpp_0.12.3 RColorBrewer_1.1-2 formatR_1.2.1
[4] nloptr_1.0.4 plyr_1.8.3 iterators_1.0.8
[7] tools_3.2.3 rpart_4.1-10 digest_0.6.9
[10] lme4_1.1-10 evaluate_0.8 nlme_3.1-122
[13] gtable_0.1.2 mgcv_1.8-9 Matrix_1.2-3
[16] foreach_1.4.3 yaml_2.1.13 parallel_3.2.3
[19] SparseM_1.7 gridExtra_2.0.0 cluster_2.0.3
[22] stringr_1.0.0 MatrixModels_0.4-1 stats4_3.2.3
[25] nnet_7.3-11 foreign_0.8-66 rmarkdown_0.9.2
[28] latticeExtra_0.6-26 minqa_1.2.4 reshape2_1.4.1
[31] car_2.1-1 magrittr_1.5 scales_0.3.0
[34] codetools_0.2-14 htmltools_0.3 MASS_7.3-45
[37] splines_3.2.3 pbkrtest_0.4-6 colorspace_1.2-6
[40] labeling_0.3 quantreg_5.19 stringi_1.0-1
[43] acepack_1.3-3.3 munsell_0.4.2

This manuscript was compiled from R code and markdown text with knitr and pandoc.

References:

1. Ho TH, Camerer CF, Chong J-K. Self-tuning experience weighted attraction learning in games. Journal of Economic Theory. 2007;133: 177–198. doi:10.1016/j.jet.2005.12.008

2. Camerer CF, Ho T-H. Behavioral game theory experiments and modeling. 2013; Available: http://faculty.haas.berkeley.edu/hoteck/papers/Camerer-Ho-Book.pdf

3. Camerer C, Hua Ho T. Experience-weighted attraction learning in normal form games. Econometrica. 1999;67: 827–874. doi:10.1111/1468-0262.00054

4. Janssen MA, Ahn T-K. Learning, signaling, and social preferences in public-good games. Ecology and society. 2006;11: 21. Available: http://www.cs.sfu.ca/~lshia/personal/econ/papers/janssenAhn1.pdf

5. Saltelli A, Annoni P. How to avoid a perfunctory sensitivity analysis. Environmental Modelling & Software. 2010;25: 1508–1517. doi:10.1016/j.envsoft.2010.04.012

6. Thiele JC, Kurth W, Grimm V. Facilitating parameter estimation and sensitivity analysis of agent-based models: A cookbook using NetLogo and r. JASSS. 2014;17: 11.

7. Beachkofski B, Grandhi R. Improved distributed hypercube sampling. 43rd AIAA/ASME/ASCE/AHS/ASC structures, structural dynamics, and materials conference. American Institute of Aeronautics; Astronautics; 2002. Available: http://arc.aiaa.org/doi/abs/10.2514/6.2002-1274

8. Carnell R. Lhs latin hypercube samples [Internet]. 2012. Available: http://cran.r-project.org/web/packages/lhs/index.html

9. Pujol G, Iooss B, Lemaitre AJ with contributions from P, Gilquin L, Gratiet LL, Touati T, et al. Sensitivity: Sensitivity analysis [Internet]. 2014. Available: http://cran.r-project.org/web/packages/sensitivity/index.html

10. Saltelli A, Chan K, Scott EM. Sensitivity analysis. 1 edition. Chichester: Wiley; 2009.
